# Optimizing treatment protocols for spinal manipulative therapy: study protocol for a randomized trial

**DOI:** 10.1186/s13063-018-2692-6

**Published:** 2018-06-04

**Authors:** Julie M. Fritz, Jason A. Sharpe, Elizabeth Lane, Doug Santillo, Tom Greene, Gregory Kawchuk

**Affiliations:** 1 College of Health, University of Utah, 520 Wakara Way, Salt Lake City, UT 84108 USA; 2 Department of Physical Therapy & Athletic Training, University of Utah, 520 Wakara Way, Salt Lake City, UT 84108 USA; 3 Department of Internal Medicine and Director, Population Health Research Study Design and Biostatistics Center, School of Medicine, University of Utah, 295 Chipeta Way, Salt Lake City, UT 84132 USA; 4 Department of Physical Therapy, Faculty of Rehabilitation Medicine, University of Alberta, 3-44 Corbett Hall, Edmonton, AB T6G 2G4 Canada

**Keywords:** Low back pain, Spinal manipulative therapy, Multiphase optimization, Factorial design

## Abstract

**Background:**

Low back pain is a common and costly condition. Spinal manipulative therapy (SMT) is a treatment supported in some guidelines, although most clinical trials examining SMT report small effect sizes. Enhancing the effects of SMT requires an understanding of underlying mechanisms and a systematic approach to leverage understanding of mechanisms to create more effective treatment protocols that are scalable in clinical practice. Prior work has identified effects on spinal stiffness and lumbar multifidus activation as possible mechanisms. This project represents a refinement phase study within the context of a multi-phase optimization strategy (MOST) framework. Our goal is to identify an optimized SMT treatment protocol by examining the impact of using co-intervention exercise strategies that are proposed to accentuate SMT mechanisms. The optimized protocol can then be evaluated in confirmation phase clinical trials and implementation studies.

**Methods:**

A phased, factorial randomized trial design will be used to evaluate the effects of three intervention components provided in eight combinations on mechanistic (spinal stiffness and multifidus muscle activation) and patient-reported outcomes (pain and disability). All participants will receive two sessions then will be randomly assigned to receive six additional sessions (or no additional treatment) over the next three weeks with factorial combinations of additional SMT and exercise co-interventions (spine mobilizing and multifidus activating). Outcome assessments occur at baseline, and one week, four weeks, and three months after enrollment. Pre-specified analyses will evaluate main effects for treatment components as well as interaction effects.

**Discussion:**

Building on preliminary findings identifying possible mechanisms of effects for SMT, this trial represents the next phase in a multiphase strategy towards the ultimate goal of developing an optimized protocol for providing SMT to patients with LBP. If successful, the results of this trial can be tested in future clinical trials in an effort to produce larger treatment benefits and improve patient-centered outcomes for individuals with LBP.

**Trial registration:**

ClinicalTrials.gov, NCT02868034. Registered on 16 August 2016.

**Electronic supplementary material:**

The online version of this article (10.1186/s13063-018-2692-6) contains supplementary material, which is available to authorized users.

## Background

Low back pain (LBP) is a major public health problem [1]. An estimated 60–80% of individuals experience an episode during their lifetime and LBP is among the most common reasons prompting a healthcare visit in the United States [2]. It is therefore not surprising that LBP imposes significant economic burden. Low back pain is the third costliest medical condition in the United States, behind only diabetes and heart disease; and costs have been increasing at the second fastest rate over the past ten years [3]. Despite the resources spent on management, LBP prevalence rates have been increasing [4], with rates of opioid prescribing for LBP rising even more rapidly [5]. These circumstances make the development and broad implementation of effective non-pharmacologic treatments an urgent priority [6].

Practice guidelines and systematic reviews identify several non-pharmacologic LBP treatments with some evidence of benefit often including spinal manipulative therapy (SMT) [7, 8]. While SMT is recommended in many guidelines, effect sizes for SMT are modest for patient-centered outcomes of pain and disability [9–11], leading other reviews to conclude the benefits of SMT are not clinically meaningful [12, 13]. A partial explanation for these equivocal recommendations is that SMT has been provided in clinical trials with highly variable protocols. Some studies have examined SMT as a unimodal strategy, while others have used different co-interventions including thermal modalities, soft tissue techniques, and various forms of exercise [14]. The optimal protocol for providing SMT treatment is currently not established.

The high degree of variability in clinical protocols may be partly attributable to the lack of understanding about the mechanisms through which SMT may provide clinical benefit [15]. Research has described the clinical presentation of individuals with LBP who are more likely to respond to SMT including more acute symptoms and absence of symptoms extending into the leg(s) [16, 17]. Optimizing treatment protocols, however, requires an understanding of the underlying reasons why some individuals respond to SMT while others do not. If the underlying mechanisms through which SMT exerts clinical benefit were understood, protocols could be developed to optimize these mechanisms through co-interventions designed to impact the same pathways.

There is a sizable body of literature documenting various physiologic effects that occur with the application of SMT [18]. Until recently, there has been little work to identify which, if any, of these effects relate to clinical benefit and which are unrelated phenomena. Prevailing theories on the mechanisms of SMT have historically focused on two primary effects resulting from SMT: (1) biomechanical effects on spinal kinetics and stiffness characteristics [19–21]; and (2) neuro-physiologic effects on primary afferent neurons leading to altered motor neuron excitability [18, 22]. Support for these theories comes from studies documenting that SMT can alter spinal stiffness [23–27] and that afferent stimulation from SMT impacts reflex motor activity and moto-neuron excitability [23, 28–32]. It remains uncertain if these effects represented mechanisms of therapeutic benefit or epiphenomena unrelated to clinical outcomes.

**Fig. 1:**
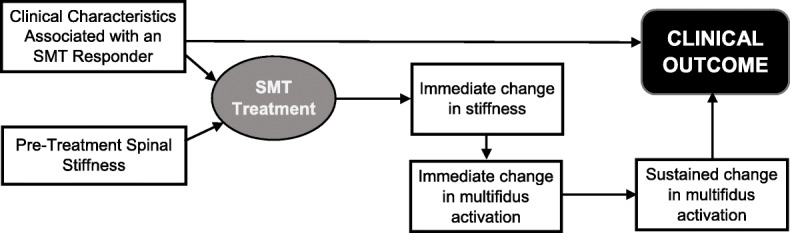
Conceptual model of underlying mechanisms of SMT based on preliminary research [33]

We have conducted preliminary work investigating the role of changes in spinal stiffness and deep trunk muscle (i.e. transversus abdominus, internal oblique, and lumbar multifidus) activation [33–35]. These studies have indicated that individuals with LBP who are clinical responders to SMT treatment are characterized by immediate decreases in spinal stiffness and improvement in activation of the lumbar multifidus muscle sustained over one-week follow-up periods. Individuals with LBP who do not benefit clinically from SMT did not display this pattern of stiffness and multifidus muscle activation changes [35] and changes to other deep trunk muscles also appeared unrelated to responsiveness to SMT [33–36]. This body of research resulted in a model of explaining the mechanisms underlying the clinical benefits of SMT (Fig. 1) that serves as the conceptual basis for this study.

Uncovering the mechanisms and optimizing the protocol for providing SMT will help advance its role as a non-pharmacologic option for patients with LBP. Based on our preliminary work and resultant conceptual model, exercises designed specifically to enhance spine mobility and lumbar multifidus muscle activation may enhance the effects of SMT and optimize clinical outcomes. In addition to co-interventions, the optimal dosage of SMT is not established. Research examining dose-response relationships for SMT have examined the treatment without any co-interventions and have failed to identify an optimal dose [37, 38].

A traditional approach to evaluating this proposed multi-component protocol combining SMT with different types of exercise and varying SMT dose would use a parallel-group randomized trial design with protocol revisions and subsequent randomized trials based on the results [39]. A particular shortcoming of this approach is the inability to test the effects of individual components and interactions among components of a multi-component treatment protocol. Within a multi-component protocol, it is possible that some intervention components actively contribute to improved outcomes, while others may be non-contributory or even detract from beneficial effects.

The Multiphase Optimization Strategy (MOST) has been described as an alternative framework for achieving the goal of optimizing a multi-component intervention through a rigorous, multi-phased strategy [40]. From a MOST perspective, optimization begins with a screening phase during which the most promising treatment components are identified and grounded within a theoretical framework. The second phase is used to refine component dosage and combinations and explore differences based on moderating effects of participant characteristics. The final phase involves confirmation of the benefits of the optimized intervention typically using a randomized trial design [39, 41].

**Fig. 2:**
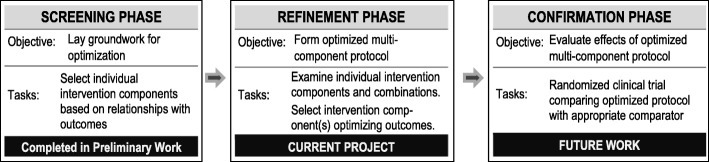
Phases of Multiphase Optimization Strategy (MOST) as applied to development of optimized SMT research [40]

We place the current study within the MOST framework as a refinement phase project (Fig. 2). Our prior work has identified mechanisms through which SMT may exert an impact on clinical outcomes for patients with LBP. This work provides a scientific rationale to select exercise co-interventions that act along the same mechanistic pathways as components that may optimize an SMT treatment program. This study uses a factorial design to permit evaluation of both main effects of three individual treatment components (SMT dose, spine mobilizing exercise, and lumbar multifidus activating exercises) and interaction effects towards the goal of identifying an optimized SMT protocol that can be evaluated in future randomized trials. The overall goal of this project is therefore to define an optimized SMT treatment protocol. The primary aim of the current study is to evaluate these three SMT treatment components and their effect on SMT mechanistic outcomes (spinal stiffness and multifidus muscle activation) and patient-centered clinical outcomes (disability and pain). Secondary aims will explore the moderating effects of baseline patient characteristics and early treatment response.

## Methods

### Study design and rationale

**Fig. 3:**
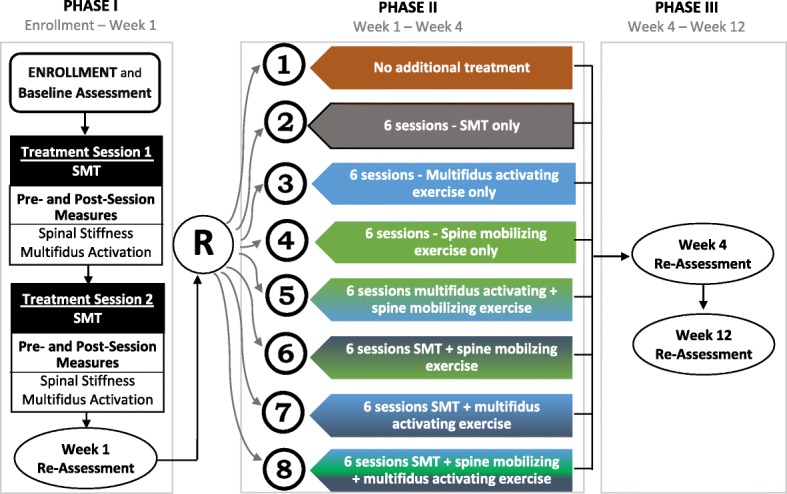
Study design showing three phases and four assessments. Each assessment includes PROs, spinal stiffness, and multifidus activation measures

This project will use a phased, factorial design examining three intervention components (SMT dose, spine mobilizing exercise, and multifidus activating exercise) provided in eight different combinations following provision of two SMT treatment sessions. The research design is outlined in Fig. 3. To begin, all participants will receive two sessions of SMT treatment (phase I) after which the one-week re-assessment will be conducted. At the one-week assessment, participants will be randomly assigned to one of eight phase II treatment groups. An aspect of the one-week re-assessment will categorize participants as treatment responders or non-responders based on a previously validated threshold of 50% reduction in disability [42]. Randomization will be stratified by responder status. Phase II treatment will occur across three weeks followed by four- and 12-week re-assessments. The CONSORT extension for non-pharmacological interventions (Additional file 1) and the Standard Protocol Items: Recommendations for Interventional Trials (SPIRIT) Checklist for the implementation of study protocols (Additional file 2) were followed in the development of the study and protocol report.

All participants will receive two SMT sessions as phase I treatment to allow replication of our conceptual model (Fig. 1) in a new LBP cohort. This design also permits examination of the moderating effects of early clinical response on longer-term outcomes. In our prior studies 35–45% of individuals with LBP were responders to the two-session SMT protocol [16, 33, 35]. What is unknown is the persistence of the improvement observed in early responders and whether persistence can be augmented through additional SMT and/or co-interventions. Likewise, it is unknown if early non-responders can be converted to responders with additional SMT and/or co-interventions. Following the two SMT sessions, we will randomly assign participants to receive six additional sessions (or no additional treatment) provided over three weeks with factorial combinations of additional SMT and exercise co-interventions (spine mobilizing and multifidus activating). We chose six additional sessions (twice weekly for three weeks) based on work related to SMT dose-response reporting only modest difference in clinical outcomes in subjects receiving 9–12 sessions relative to 3–6 SMT sessions without co-interventions [37, 38]. The factorial design allows evaluation of main effects of each component and interaction effects.

### Study participants

The eligibility criteria were designed to recruit a sample of individuals with non-specific LBP without contraindications to SMT or the co-interventions used in the study. The criteria are consistent with those from our prior research. Reason(s) for ineligibility will be monitored and recorded so that eligibility and consent rates can be determined. Eligibility for participation requires each of the following criteria be met:

- Pain between the 12th rib and buttocks with or without symptoms into one or both legs, which, in the opinion of the examiner, originate from the lumbar region.
- Age 18–60 years at the time of enrollment
- Oswestry disability score ≥ 20%
- No prior surgery to the lumbosacral spine
- Not currently pregnant
- Not currently receiving mind–body or exercise treatment for LBP from a healthcare provider (e.g. chiropractic, physical therapy, massage therapy, etc.)
- No neurogenic signs on clinical examination including diminished myotomal strength, muscle stretch reflexes or sensation, or positive straight leg raise sign
- No known serious spinal pathology (e.g. spinal tumor, fracture, infectious disorder, osteoporosis, or other bone demineralizing condition) or suspicion of serious pathology based on red flags noted in the general medical screening.

### Study measures

**Fig. 4:**
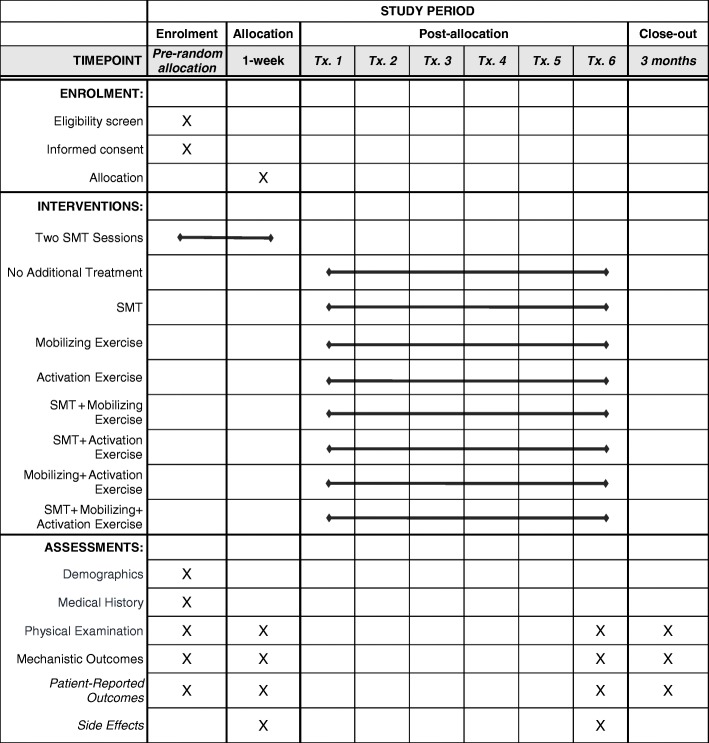
The Vertetrack device positioned over a participant to assess spinal stiffness

Assessments will be conducted at baseline, after one week (completion of treatment phase I), four weeks (completion of treatment phase II), and 12 weeks. The one-week examination will permit assessment of change in stiffness and activation occurring in the short term using the same time frame as in our validation studies and clinical assessment for responder status. The four-week examination will evaluate short-term effects of different SMT protocols. The three-month assessment will evaluate persistence of these effects. All assessments involve physical examination and collection of mechanistic and patient-reported outcomes (PROs). Relevant participant demographic and LBP history variables will be collected at enrollment. Data from study sites will be collected and consolidated via REDCap (Research Electronic Data Capture) with appropriate quality checks on data entry [43] (Fig. 4).

#### History and physical examination

History and physical examination will include clinical characteristics associated with an SMT response in prior studies including the duration of current LBP symptoms, presence of any symptoms (pain, numbness, tingling) extending below the knee in the past 72 h, and spinal mobility assessed with manually applied posterior-anterior force assessed with the subject prone [16]. We will evaluate multifidus activation using the lift test [44]. Lumbar spine and hip range of motion will be evaluated using validated measurement techniques [45].

#### Patient-reported outcomes

PROs were selected to reflect outcomes that are most meaningful to individuals with LBP and to quantify participant’s beliefs and attitudes about LBP. The primary PROs are the Oswestry Disability Questionnaire (ODQ) and Numeric Pain Rating Scale (NPRS). The ODQ is a LBP-specific measure of function for patients with LBP assessed on a 0–100 scale, with lower numbers indicating less LBP-related disability. We will use a 0–10 NPRS (“0” no pain and “10” worst imaginable pain) to assess LBP intensity. Both the ODQ and NPRS have high test–retest reliability, good construct validity, and responsiveness to change for patients with LBP [46–48].

The ODQ score will be used to determine responder status as a binary outcome at each follow-up. Based on prior research [42], a participant whose ODQ score is improved (i.e. decreased) by at least 50% relative to the baseline ODQ score is a treatment responder, while those with < 50% improvement are defined as non-responders. The 50% threshold is a more stringent definition of treatment responder than a threshold corresponding to the minimum clinically important difference which is generally defined as at least 30% improvement from baseline [49].

We will assess pain catastrophizing and fear-avoidance beliefs about LBP because these constructs have been found to predict chronicity and mediate treatment effects among patients with LBP [50–53]. The Fear Avoidance Beliefs Questionnaire (FABQ) [54] will be used to measure patients’ beliefs about how physical activity (FABQPA) and work (FABQW) may affect their LBP and perceived risk for re-injury. The Pain Catastrophizing Scale (PCS) will be used to measure the extent to which people catastrophize in response to pain [55]. These variables may serve as covariates in the analyses to control for psychosocial risk factors.

#### Side effects and adverse events

At the one- and four-week follow-ups, participants will complete a questionnaire about side effects they may have experienced with study treatment [56]. The questionnaire asks about commonly described side effects (muscle spasm, radiating discomfort, etc.) and allows participants to identify anything they perceive as an adverse symptom resulting from treatment. For each side effect identified, participants indicate the time of onset relative to their last treatment session (< 1 h, 1–24 h, or > 24 h), the severity (light, mild, moderate, or severe), and duration (< 1 h, 1–24 h, or > 24 h) of the symptom. Other adverse events identified by any member of the research team will be recorded and reported as appropriate to institutional review boards and study monitoring groups.

#### Mechanistic outcomes

**Fig. 5:**
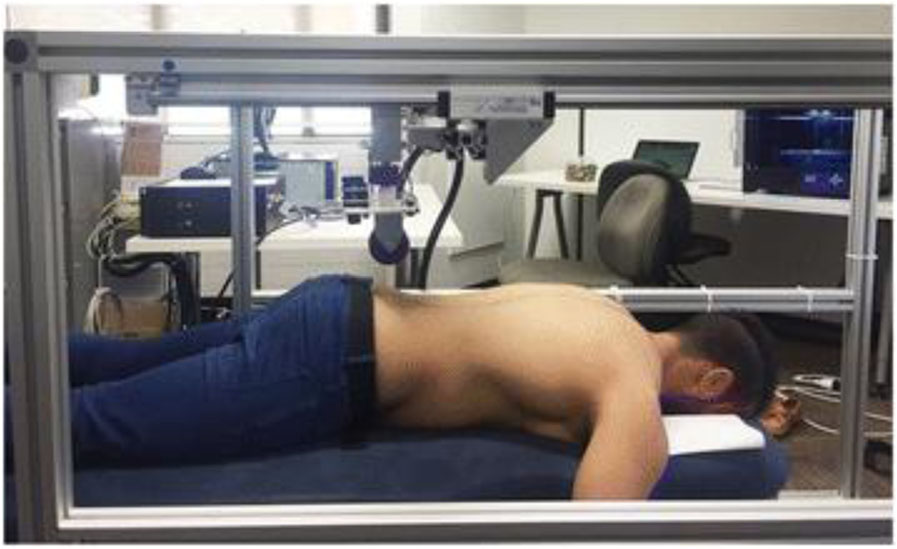
The preferred SMT technique to be used in this study

Mechanistic outcomes will assess the constructs underlying the therapeutic effects of SMT in our conceptual model including spinal stiffness and lumbar multifidus muscle activation (Fig. 1). The VerteTrack device will be used to measure lumbar spine stiffness. In this measure, the VerteTrack frame and indenter apparatus are positioned over the prone-lying participant. The indenter apparatus consists of a rod suspended within a linear bearing to permit near-frictionless vertical translation in conjunction with an indentation roller comprising two circular plastic disks (Fig. 5). Force transfer to the participant occurs via the rod loaded with mass of increasing magnitude applied through the indentation roller. The rollers straddle the participant’s spinous processes to provide a rolling contact point for the application of vertical loads. The indenter houses a sensor to provide continuous, real-time quantification of the bulk deformation of any spinal region for a given mass over a defined trajectory. Further details on the VerteTrack device operations have been published [57]. Force displacement curves for the lumbar spine are used to calculate terminal stiffness by dividing the maximum applied force by the maximal displacement expressed in N/mm.

Multifidus activation will be measured with brightness-mode ultrasound images using a Sonosite MicroMaxx (Sonosite Inc. Bothell, WA, USA) and a 60-mm, 2–5 MHz curvilinear array. Measures are taken with the participant prone with neck in neutral and arms overhead at about 120° of shoulder abduction. The ultrasound transducer is placed just lateral to the spinal midline and angled medially until a parasagittal view of the multifidus at the L_4_-L_5_ and L_5_-S_1_ levels is obtained. Images are acquired at each level with the multifidus at rest and during submaximal contraction elicited by the participant lifting the contralateral arm about 2 in. while holding a weight proportional to body weight. This procedure results in ~ 30% maximum voluntary isometric multifidus contraction. Offline multifidus thickness measures are obtained from determining the distance between the posterior-most aspect of the facet joint inferiorly and the plane between the multifidus and thoracolumbar fascia superior. Muscle activation is calculated as the change in thickness at rest and submaximal contraction (Thickness_contract_–Thickness_rest_) /Thickness_rest_). Research has shown these measures of multifidus activation have good concurrent validity compared to EMG activity of the muscle [58].

### Recruitment and allocation

Participants will be recruited at two sites: the University of Utah and University of Alberta. At each site, potential participants will be recruited from individuals seeking healthcare within these systems as well as from general public advertising. Interested individuals will meet with a member of the research team and those who provide informed consent will begin phase I treatment with all participants receiving two SMT sessions. After completion of the two treatment sessions, each participant’s responder status will be determined at the one-week follow-up. Randomization to one of eight phase II treatment groups will occur after the one-week follow-up is complete. Blocked randomization with block sizes of four or six will be used. The randomization schedule will be prepared before participant enrollment in the study by project statisticians. Randomization will be stratified based on site (University of Utah or Alberta) and responder status after two SMT sessions.

### Blinding

Participants in this study cannot be blinded to treatment. Because our purpose is to optimize protocols, not to evaluate SMT efficacy, we are not using placebos or attempts to balance clinician time. Randomization assignment will not be revealed until the baseline examination, the first two SMT sessions, and the one-week follow-up are complete to reduce potential bias and maintain blinding of the participant, clinicians, and researchers to the eventual group assignment. Randomization allocation will be done using RedCap in order to conceal sequence from participants and researchers. The four- and 12-week follow-up assessments will be performed by a researcher blinded to participants’ treatment group. Clinicians providing treatment after randomization cannot be blinded. The use of standard protocols and compliance audits throughout the project will minimize potential bias related to differential treatment application.

### Treatments

Treatments used in this study include SMT, spine mobilizing exercise, and multifidus activating exercise. The same SMT protocol will be used in phase I and II treatment phases. The exercise treatments will be used during treatment phase II only. Participants will receive all prescribed sessions unless participant requests to discontinue. Outcome measurements will continue if a participant discontinues treatment sessions. All treatments will be provided by credentialed providers (either licensed chiropractors or physical therapists).

#### Spinal manipulative therapy

**Fig. 6:**
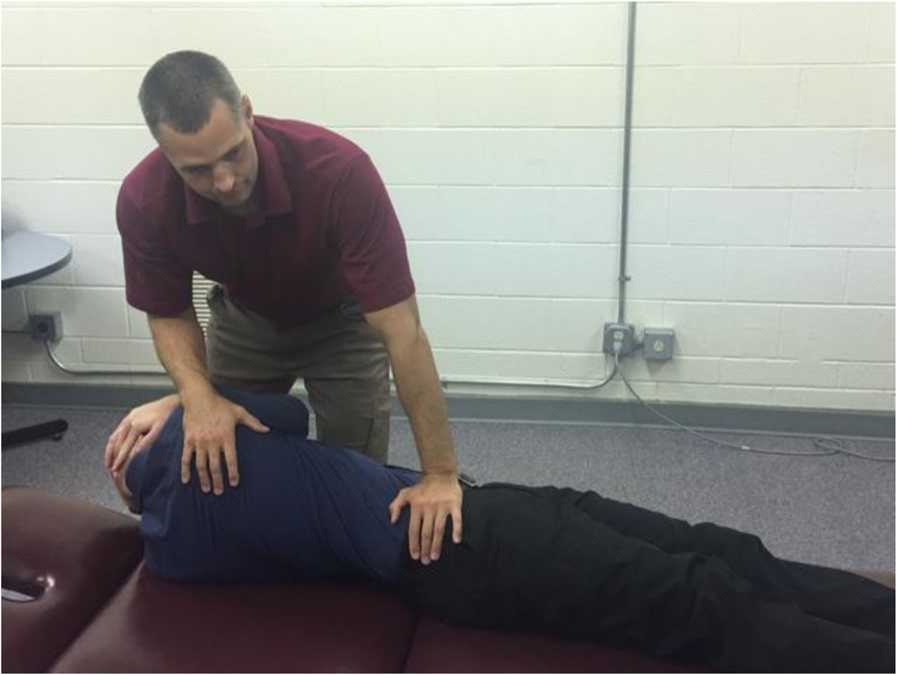
Study schedule based on the Standard Protocol Items: Recommendations for Interventional Trials (SPIRIT) guidelines

All SMT treatment sessions will be provided using protocols applied in our prior work investigating clinical outcomes and mechanisms of effect [16, 33, 35, 59, 60]. All SMT sessions will begin with a brief assessment by the clinician followed by treatment. The preferred SMT technique is performed with the participant supine. The clinician stands opposite the side to be manipulated and side-bends the participant. The side to be manipulated is the side identified as more painful. The participant interlocks their fingers behind the head. The clinician rotates the participant and delivers a high-velocity, low-amplitude (HVLA) thrust to the anterior superior ilinferior direction/inferior direction (Fig. 6). The clinician notes if a cavitation (i.e. a “pop”) occurred and, if noted, the SMT treatment is complete. If no cavitation occurs, the participant is repositioned and SMT is performed again. If no cavitation occurs on the second attempt, the clinician will manipulate the opposite side. A maximum of two attempts per side is permitted. Our prior research found no difference in outcome between this preferred SMT procedure and an alternative side-lying technique [60]. We will permit substitution with the side-lying technique based on participant preference or comfort.

#### Spine mobilizing exercise

Participants randomized to receive mobilizing exercises during phase II will be instructed in a program of repeated movements progressing into end-ranges of spinal flexion and/or extension based on principles described by McKenzie [61] and shown in past studies to improve ROM and reduce spinal stiffness [62, 63]. The basic protocol for progression of mobilizing exercises is outlined in Table 1. Participants will be instructed in mid-range exercises and will be further assessed for a directional preference. A directional preference is present if movement in a particular direction decreases LBP intensity or causes symptoms to centralize towards the midline [64]. If a participant has a directional preference he or she will be prescribed exercises specifically in that direction along with mid-range exercise. Otherwise the participant will be assigned exercises moving into either flexion or extension based on the clinician’s discretion. Participants will perform their prescribed exercises following SMT at treatment sessions and will be instructed to perform the exercises daily on other days. Prescribed exercises and participant compliance with assigned exercises will be recorded at each session.

**Table 1:** Spine mobilizing exercise protocol

Activity	Description	Initial dose	Goals for progression
Mid-range spinal mobility exercises	• Supine pelvic tilts to promote lumbar flexion/extension • Quadruped rocking into lumbar flexion/extension • Supine to side lying rotational mobilizations • Sitting rotational mobilizations • Flexion/Extension	10–20 repetitions each direction performed daily (each exercise)	Full, pain-free ROM, progress to 40 repetitions throughout the day
Exercises specifically into spinal flexion	• Supine pelvic tilt • Quadruped rocking into lumbar flexion • Double knee-to-chest while supine • Standing flexion • Seated flexion • Self-mobilization into flexion	10–20 repetitions performed daily (prescribe 2 exercises)	Full, pain-free ROM, progress to 40 repetitions throughout the day
Exercises specifically into spinal extension	• Supine pelvic tilt • Quadruped rocking into lumbar flexion • Supported on elbows while prone 30 s • Prone press-ups to extended elbows • Prone press-ups to extended elbows with exhale • Extension while standing • Extension in standing with self over pressure	10–20 repetitions performed daily (prescribe 2 exercises)	Full, pain-free ROM, progress to 40 repetitions throughout the day

#### Multifidus activating exercise

Participants randomized to receive multifidus exercises will begin with isometric multifidus contractions in different positions with clinician feedback and exercises to isometrically co-contract the multifidus and deep abdominal muscles. These exercises have been shown to be effective for activating the multifidus muscle [65]. Participants will also perform lumbar extensor strengthening exercises shown to produce 20–50% of multifidus maximum voluntary contraction (Table 2) [66, 67]. This dose is adequate to enhance multifidus activation, without imposing high loads that may exacerbate LBP. Participants will continue to perform isometric exercises throughout treatment. Prescribed exercises and participant compliance with assigned exercises will be recorded at each session.

**Table 2:** Multifidus activation exercise protocol

Activity	Description	Initial dose	Goals for progression
Preferential, isometric multifidus activation exercises	• Isolated multifidus contraction while prone, seated, standing • Isolated co-contraction of multifidus and deep abdominals in sitting, standing	5 repetitions, 10-s hold with normal breathing	Progress towards 10 repetitions, 10-s hold, perform 2–3× daily
General lumbar extensor and multifidus activation exercises	• Quadruped single arm raises	10 lifts, 5-s hold each arm	Progress towards 20 lifts, add arm + leg lift
	• Side-support exercise	10 repetitions, 5-s hold each side	Progress towards 20 repetitions
	• Bridging while hook-lying	10 repetitions, 5-s hold	Progress towards 20 repetitions
	• Prone single leg lift	10 lifts, 5-s hold each leg	Progress towards 20 lifts, add arm + leg lift
	• Prone trunk lift	10 lifts, 5-s hold	Progress towards 20 lifts

### Statistical analysis

Our primary hypothesis is that one or more combination of treatment components will optimize improvement in SMT mechanistic effects (reduction in spinal stiffness and improvement in multifidus activation) and improvement in patient-centered outcomes (ODQ and NPRS). Our secondary hypothesis is that responder status after two sessions will moderate mechanistic and patient-centered outcomes.

The effects of the treatment components (A additional SMT; B multifidus activation; C mobilizing exercises) on each outcome will be evaluated using linear mixed models to relate mean levels of each outcome at one week, four weeks, and three months to indicator variables to represent the main effects (A, B, C), pairwise interactions (A × B, A × C, B × C), and the three-way interaction (A × B × C). The one-week assessment, which occurs just before randomization, will serve as the baseline for these analyses. An unstructured covariance matrix will be used to account for correlation of serially measured outcome scores. By using an unstructured covariance matrix, the model will constitute a special case of a general linear mixed model which avoids imposing specific assumptions concerning distribution of random effects. This modeling approach is recommended in randomized trials when the number of assessments is small [68].

Restricted maximum likelihood estimation will be used for estimation of parameters and associated confidence intervals (CI) [69] for the following quantities for each outcome at four- and 12-week assessments: (1) main effects evaluating effects of each of the three treatment components while averaging over the levels of the other two components; (2) three pairwise interactions (A × B, A × C, B × C) evaluating if the effect of a component differs between levels of one of the other components while averaging over the levels of the third component. Pairwise interactions will inform whether the effects of each intervention pair are additive, synergistic, or antagonistic; and (3) three-way interaction (A × B × C) evaluating if each pairwise interaction differs depending on the third component. To account for co-primary outcomes each of the hypothesis tests noted above will be performed with two-sided α = 0.025 and CIs will be constructed using a confidence coefficient of 0.975. The indicated comparisons at four weeks will be given primary emphasis in evaluating the effects of each treatment component. Comparisons at 12 weeks will evaluate persistence of effects.

In order to assess which treatment component combinations optimize outcomes, we will first simplify the fully saturated factorial analysis of variance model by comparing the Bayes Information Criterion (BIC) among all possible models including different combinations of main effects, pairwise interactions, and the three-way interaction which satisfy the hierarchical consistency constraint that the main effects corresponding to each term in a pairwise interaction are retained in models with pairwise interactions, and all component main effects and pairwise interactions are retained when considering the three-way interaction. This will result in a more parsimonious model to increase statistical power. Then, using the simplified model, for each outcome we will use a simulation approach [70] to derive simultaneous 97.5% CIs for all comparisons of estimated mean outcome under each possible treatment combinations. After ordering the treatment combinations in accordance with the observed mean outcome, the simultaneous CIs will be used to identify which combinations are statistically indistinguishable from the optimum treatment, thus identifying a set of candidate options for the best combination of treatment components. This process will be applied for both mechanistic and patient-centered outcomes.

We will explore responder status after two SMT sessions as a possible effect moderator by adding a main effect for responder status and interaction terms between responder status and the indicator variables for the treatment components which are retrained in the final simplified models for the different outcomes developed using the BIC criteria for each of the outcomes for the primary aims. Statistically significant interactions between responder status and main effect and/or interaction terms between treatment components will be interpreted as suggesting effect moderation. Recognizing that tests for interactions have limited statistical power, we will also fit the simplified models developed in the primary aim separately under the presence and absence of each factor (dichotomizing using a median split for continuous factors) and graphically display the estimated treatment effects at both levels of responder status. Recognizing the potential for lower statistical power, the results of this aim will be interpreted as exploratory.

### Sample size and missing data

We estimated standard deviations and pre-post correlations for each outcome based on our prior work [33]. Assuming a sample size of 280 and 92% retention to four weeks, Table 3 displays minimum detectable effect sizes for: (1) main effects of each components; (2) pairwise interactions between two components; (3) comparison of mean outcome between two levels of one component at a fixed level of another component; (4) main effects of the three components in subgroup analyses involving half the participants; and (5) pairwise interactions between two components in subgroup analyses with half the participants. Our sample size provides at least 80% power to detect the MCIDs or hypothesized effect sizes for the main effects of each component for all four outcomes and for analyses of main effects in subgroups and conditional comparisons for each outcome except global stiffness. Power is more limited for secondary aims, such as pairwise interactions within subgroups.

**Table 3:** Assumptions and detectable effect sizes informing sample size for the project

	Assumptions for outcome measures			
	Stiffness (N)	Multifidus activation (mm)	Oswestry	Numeric Pain Rating
*Assumption*				
Mean (SD)	5.55 (1.60)	2.60 (0.124)	24.3 (14.9)	5.06 (2.12)
Pre-post score correlation	0.80	0.70	0.70	0.45
MCID or hypothesized effect size from prior work	0.40	0.074	6.0	2.0
*Type of effect*	Detectable effect size			
Main effect	0.37	0.034	4.12	0.76
Pairwise interaction	0.74	0.068	8.24	1.53
Conditional comparison	0.53	0.048	5.83	1.08
Main effect in 50% of participants	0.53	0.045	5.83	1.08
Interaction in 50% of participants	1.05	0.091	11.65	2.16

Because the analyses of the longitudinal models will be based on restricted maximum likelihood estimation, statistical inferences will remain valid so long as missing data follow a missing at random structure [69]. We do not believe missing data will lead to substantial bias in our primary evaluation of protocol components. However, we will compare participant characteristics between subgroups with missing and non-missing data at four weeks and if substantial deviations are detected or the rate of missing data is greater than expected, multiple imputation will be applied using comprehensive imputation models which include auxiliary variables to account for additional predictors of missingness and/or the values of the outcome variables [71].

### Trial and data monitoring

Trial supervision includes a steering committee composed of the investigators and representatives of the funding agency (National Institutes of Health). A separate Data Safety and Monitoring Committee comprising three external members with expertise in SMT, LBP, and clinical trials will also provide at least annual review of study and any safety issues as necessary. Protocol modifications will be reported to these bodies and recorded in trial registry.

## Discussion

Moving beyond the modest effect sizes for LBP treatments including SMT requires evidence informing optimal treatment protocols. Optimized protocols can then be tested in clinical trials. The MOST framework provides a multi-phase strategy to optimize an intervention protocol. Based on our prior work, we are examining SMT provided in various dosages with exercise co-interventions designed to accentuate SMT’s effects of spinal stiffness and multifidus muscle activation. Our factorial design will permit efficient examination of these treatment components and their interactions on clinical as well as mechanistic outcomes. We will also consider the moderating effect of early response to a brief (two-session) SMT protocol. This strategy will address an important clinical question related to the interpretation of early response to SMT and the extent to which it should influence subsequent treatment decisions.

This project provides innovation in several areas of critical importance for future studies of SMT. First, our project will be the first that seeks to optimize SMT treatment protocols for patients with LBP that is guided by a validated model explaining the mechanisms underlying the therapeutic effects of SMT. The project investigates clinically relevant and potentially scalable SMT protocol components. Our project examines both short- and long-term outcomes. Additionally, we are grounding our work in the MOST framework described specifically for optimizing multi-component treatment protocols but not previously applied to SMT.

Our design comes with several important limitations. Randomization to co-interventions will occur after two SMT treatment sessions. In clinical practice, co-interventions typically are initiated alongside SMT instead of our phase approach. Repeated spinal stiffness and multifidus muscle activation measures may influence outcomes in a manner that is also not reflective of clinical practice. Our optimization strategy incorporates only two dosages of SMT (two or eight sessions). Our long-term assessment only extends to three months following enrollment which may not be adequate for a chronic recurrent condition like LBP. Our protocol does not permit clinicians or participants to be blinded to intervention groups.

This trial has the potential to advance understanding of the underlying mechanisms of SMT for individuals with LBP and promote the identification of SMT protocols that can be implemented in clinical trials and practice. We are placing this study within a MOST context and our goal for this phase II project is to define an optimized protocol for providing SMT using a factorial design. The optimized protocol defined in this study can then be examined in future randomized clinical trials in an effort to move beyond modest effect sizes for SMT and ultimately to enhance patient-centered outcomes for patients seeking treatment for LBP.

## Additional files

Additional file 1: 2017 CONSORT checklist of information to include when reporting a randomized trial assessing nonpharmacologic treatments (NPTs)*. (DOCX 38 kb)

Additional file 2: SPIRIT 2013 Checklist: Recommended items to address in a clinical trial protocol and related documents*. (DOC 123 kb)

## Abbreviations

FABQ: Fear avoidance beliefs questionnaire
LBP: Low back pain
MOST: Multiphase Optimization Strategy
NPRS: Numeric pain rating scale
ODQ: Oswestry disability questionnaire
PCS: Pain catastrophizing scale
SMT: Spinal manipulative therapy
