# Supplementary material for: Optimizing treatment protocols for spinal manipulative therapy: study protocol for a randomized trial
Source: Trials. 2018 Jun 4;19:306. doi: 10.1186/s13063-018-2692-6 (PMC5987587; doi:10.1186/s13063-018-2692-6)
Supplement: Supplementary file 1 — 2017 CONSORT checklist of information to include when reporting a randomized trial assessing nonpharmacologic treatments (NPTs)*. (DOCX 38 kb) [file 13063_2018_2692_MOESM1_ESM.docx]

2017 CONSORT checklist of information to include when reporting a randomized trial assessing nonpharmacologic treatments (NPTs).*

| Section/Topic Item | Checklist item no. | CONSORT item with extension for NPT trials | Manuscript Page/Line Numbers |
| --- | --- | --- | --- |
| Title/abstract |  |  |  |
|  | 1a | Identification as a randomized trial in the title | Page 1 |
|  | 1b | Structured summary of trial design, methods, results, and conclusions (for specific guidance see CONSORT for abstracts) | Page 2-3 |
| Introduction |  |  |  |
| Background / objectives | 2a | Scientific background and explanation of rationale | Page 4-6 |
|  | 2b | Specific objectives or hypotheses | Page 7 / Lines 143-148 |
| Methods |  |  |  |
| Trial design | 3a | Description of trial design (e.g., parallel, factorial) including allocation ratio. When applicable, how care providers were allocated to each trial group | Page 7/Lines 151-153. (provider allocation is NA to study) |
|  | 3b | Important changes to methods after trial commencement (such as eligibility criteria), with reasons | NA |
| Participants | 4a | Eligibility criteria for participants, When applicable, eligibility criteria for centers and for care providers | Participant eligibility: Page 8/9, Lines 176-193; Provider eligibility: Page 13, Lines 312-313; Center eligibility: NA |
|  | 4b | Settings and locations where the data were collected | Page 12 / Lines 278-280 |
| Interventions | 5 | Interventions for each group with sufficient detail to allow replication, including how and when they were actually administered | Pages 13-15 |
|  | 5a | Description of the different components of the interventions and, when applicable, description of the procedure for tailoring the interventions to individual participants. | Pages 13-15, Table 1-2 |
|  | 5b | Details of whether and how the interventions were standardized. | Pages 13-15, Table 1-2 |
|  | 5c. | Details of whether and how adherence of care providers to the protocol was assessed or enhanced | Page 13, Lines 303-304 |
|  | 5d | Details of whether and how adherence of participants to interventions was assessed or enhanced | Page 15 / Lines 343-344, 354-355 |
| Outcomes | 6a | Completely defined pre-specified primary and secondary outcome measures, including how and when assessed | Page 10-12 |
|  | 6b | Any changes to trial outcomes after the trial commenced, with reasons | NA |
| Sample size | 7a | How sample size was determined, when applicable, details of whether and how clustering by providers or centers was addressed | Page 17-18 / Lines 417-427, Table 3,  clustering NA |
|  | 7b | When applicable, explanation of any interim analyses and stopping guidelines | NA |
| Randomization: |  |  |  |
| - Sequence generation | 8a | Method used to generate the random allocation sequence | Page 12-13 / Lines 284-287 |
|  | 8b | Type of randomization; details of any restriction (e.g., blocking and block size) | Page 13 / Lines 288-292 |
| - Allocation concealment mechanism | 9 | Mechanism used to implement the random allocation sequence, any steps taken to conceal sequence until interventions were assigned | Page 13 / page 296-302 |
| - Implementation | 10 | Who generated the random allocation sequence, who enrolled participants, and who assigned participants to interventions | Page 13 / Lines 290-291 |
| Blinding | 11a | If done, who was blinded after assignment to intervention (e.g, participants, providers, those assessing outcomes) and how | Page 13 / Lines 295-304 |
|  | 11b | If relevant, description of the similarity of interventions | Pages 13-15, Table 1-2 |
|  | 11c | If blinding was not possible, description of any attempts to limit bias | Page 13 / Lines 290-299 |
| Statistical methods | 12a | Statistical methods used to compare groups for primary and secondary outcomes, if applicable, details of how the clustering by providers or centers was addressed | Pages 15-17,  clustering effects are NA |
|  | 12b | Methods for additional analyses, such as subgroup analyses and adjusted analyses | Pages 15-17 |
| Results |  |  |  |
| Participant flow (a diagram is strongly recommended) | 13a | For each group, the numbers of participants who were randomly assigned, received intended treatment, and were analyzed for the primary outcome, The number of care providers or centers performing the intervention in each group and the number of patients treated by each care provider or in each center | NA |
|  | 13b | For each group, losses and exclusions after randomization, together with reasons | NA |
|  | 13c | For each group, the delay between randomization and the initiation of the intervention | NA |
|  | new | Details of the experimental treatment and comparator as they were implemented | NA |
| Recruitment | 14a | Dates defining the periods of recruitment and follow-up | NA |
|  | 14b | Why the trial ended or was stopped | NA |
| Baseline data | 15 | A table showing baseline demographic and clinical characteristics for each group | NA |
| Numbers analyzed | 16 | For each group, number of participants (denominator) included in each analysis and whether the analysis was by original assigned groups | NA |
| Outcomes and estimation | 17a | For each primary and secondary outcome, results for each group, and the estimated effect size and its precision (such as 95% confidence interval) | NA |
|  | 17b | For binary outcomes, presentation of both absolute and relative effect sizes is recommended | NA |
| Ancillary analyses | 18 | Results of any other analyses performed, including subgroup analyses and adjusted analyses, distinguishing pre-specified from exploratory | NA |
| Harms | 19 | All important harms or unintended effects in each group (for specific guidance see CONSORT for harms) | NA |
| **Discussion** |  |  |  |
| Limitations | 20 | Trial limitations, addressing sources of potential bias, imprecision, and, if relevant, multiplicity of analyses. In addition, take into account choice of comparator, lack of or partial blinding, and unequal expertise of care providers or centers in each group | Page 19-20 / Lines 466-473 |
| Generalizability | 21 | Generalizability (external validity, applicability) of the trial findings | NA |
| Interpretation | 22 | Interpretation consistent with results, balancing benefits and harms, and considering other relevant evidence | Page 20 / Lines 476-482 |
| Other information |  |  |  |
| Registration | 23 | Registration number and name of trial registry | Page 3 / Line 52 |
| Protocol | 24 | Where the full trial protocol can be accessed, if available | NA |
| Funding | 25 | Sources of funding and other support (such as supply of drugs), role of funders | Page 21 / Lines 495-501 |

**Additions or modifications to the 2010 CONSORT checklist. CONSORT = Consolidated Standards of Reporting Trials*

*†The items 5, 5a, 5b, 5c, 5d are consistent with the Template for Intervention Description and Replication (TIDieR) checklist*
